# Collective effects of long-range DNA methylations predict gene expressions and estimate phenotypes in cancer

**DOI:** 10.1038/s41598-020-60845-2

**Published:** 2020-03-03

**Authors:** Soyeon Kim, Hyun Jung Park, Xiangqin Cui, Degui Zhi

**Affiliations:** 1 Department of Pediatrics, School of Medicine, University of Pittsburgh, Pittsburgh, Pennsylvania United States; 2 Division of Pediatric Pulmonary Medicine, UPMC Children’s hospital of Pittsburgh, Pittsburgh, Pennsylvania United States; 3 Department of Human Genetics, Graduate School of Public Health, University of Pittsburgh, Pennsylvania, United States; 4 Department of Biostatistics and Bioinformatics, Emory University, Atlanta, Georgia United States; 5 Center for Precision Health, School of Biomedical Informatics, School of Public Health, University of Texas Health Center at Houston, Houston, Texas United States

**Keywords:** DNA methylation, Gene expression, Gene regulation, Cancer, Computational biology and bioinformatics, Diseases

## Abstract

DNA methylation of various genomic regions has been found to be associated with gene expression in diverse biological contexts. However, most genome-wide studies have focused on the effect of (1) methylation in *cis*, not in *trans* and (2) a single CpG, not the collective effects of multiple CpGs, on gene expression. In this study, we developed a statistical machine learning model, geneEXPLORE (gene expression prediction by long-range epigenetics), that quantifies the collective effects of both *cis*- and *trans-* methylations on gene expression. By applying geneEXPLORE to The Cancer Genome Atlas (TCGA) breast and 10 other types of cancer data, we found that most genes are associated with methylations of as much as 10 Mb from the promoters or more, and the long-range methylation explains 50% of the variation in gene expression on average, far greater than *cis*-methylation. geneEXPLORE outperforms competing methods such as BioMethyl and MethylXcan. Further, the predicted gene expressions could predict clinical phenotypes such as breast tumor status and estrogen receptor status (AUC = 0.999, 0.94 respectively) as accurately as the measured gene expression levels. These results suggest that geneEXPLORE provides a means for accurate imputation of gene expression, which can be further used to predict clinical phenotypes.

## Introduction

Many studies found the associations between DNA methylation, an essential epigenetic marker, and gene expression^1^. Methylation within the gene promoter inhibits transcription of the gene^2,3^. Methylation in the gene body can be positively correlated with the gene expression level^4^. Enhancer regions are associated with low levels of CpG methylation^5^. In addition, expression quantitative trait methylations (eQTMs) have found associations between *cis* methylation regions and gene expression^6,7^.

In cancer, hypomethylation and hypermethylation were observed at some promoters of genes^8,9^. Tumor suppressor genes are inactivated by hypermethylation in promoter regions^9^. While aberrant methylation in promoter regions mostly affects transcription in cancer, hypermethylation in gene body regions may not have a noticeable effect on transcription in cancer^10^.

Recent studies have examined the effect of methylation in *cis* enhancer regions of genes in cancer. Aran *et al*.^11^ computationally found that the association between enhancer methylation and gene deregulation in cancer was significantly stronger than the association of promoter methylation with gene deregulation, demonstrating the importance of distal methylation. Yao *et al*.^12^ inferred cancer-specific *cis* -enhancers from methylome and transcriptome analysis in multiple cancer types. However, their studies have focused on the effect of methylation *in cis* (ex. within 1 Mb from Transcription Start Site (TSS) or nearby genes from a CpG site) on gene expression.

To better understand the associations between methylation and gene expression, studying *trans* regions is critical. This is because enhancers play an important role in dysregulation of gene expression in cancer^13^, and they can be located more than a few Mb from a gene^14^. For example, a super-enhancer of the MYC gene is reported to be located 1.47 Mb from the TSS of the gene in T cell acute lymphoblastic leukemia^15^.

In addition, to fully understand the effect of distal methylation associated with gene expression, it is important to consider the collective effect of multiple associated methylations on gene expression, because multiple enhancers regulate expression of a single gene^14,16,17^. However, most statistical approaches are limited to testing a single probe and a single gene at a time, such as eQTMs and ELMER^12^, making it difficult to quantify the collective effect of CpG methylation on gene expression.

To address these issues, we developed geneEXPLORE (gene expression prediction by long-range epigenetics), a statistical machine learning method. For each gene, geneEXPLORE identifies CpG methylations, both *cis* and *trans*, that are associated with the gene expression and quantifies the collective effects of multiple CpG methylations. Based on the associated methylation probes, geneEXPLORE builds a predictive model for gene expression. We predicted expression levels of ~14,000 genes using geneEXPLORE in TCGA breast cancer data and validated the predictions in another breast cancer cohort. We also showed the applicability of geneEXPLORE method to various types of cancer. To evaluate the applicability of the gene expressions predicted by geneEXPLORE to downstream tasks, we further predicted the breast cancer phenotypes, such as breast tumor or normal status, estrogen-receptor (ER) status, 5-year survival, and breast cancer subtypes. Since the predicted gene expression represents the portion of gene expression that is associated with methylation, the present study provides a mechanistic insight into the collective effects of long-range methylation on gene expression and cancer phenotypes through gene expression using statistical models.

## Results

### gene expression prediction by long-range epigenetics (geneEXPLORE)

geneEXPLORE quantifies the collective effects of CpG methylations on gene expression by exploiting long-range regulatory elements up to the entire chromosome on which the gene is located. Because multiple distal regulatory elements interact to regulate gene expression^14,16,17^, geneEXPLORE is expected to make more accurate predictions of gene expression than the models that only use *cis-*elements such as Biomethyl^18^ and MethylXcan^19^. As gene expression is often profiled to determine clinical phenotypes, the predicted gene expression, therefore, can also be used to predict the phenotypes. The prediction accuracy of phenotypes can also indicate the collective roles of distal methylations on the phenotypes through gene expression.

**Figure 1:**
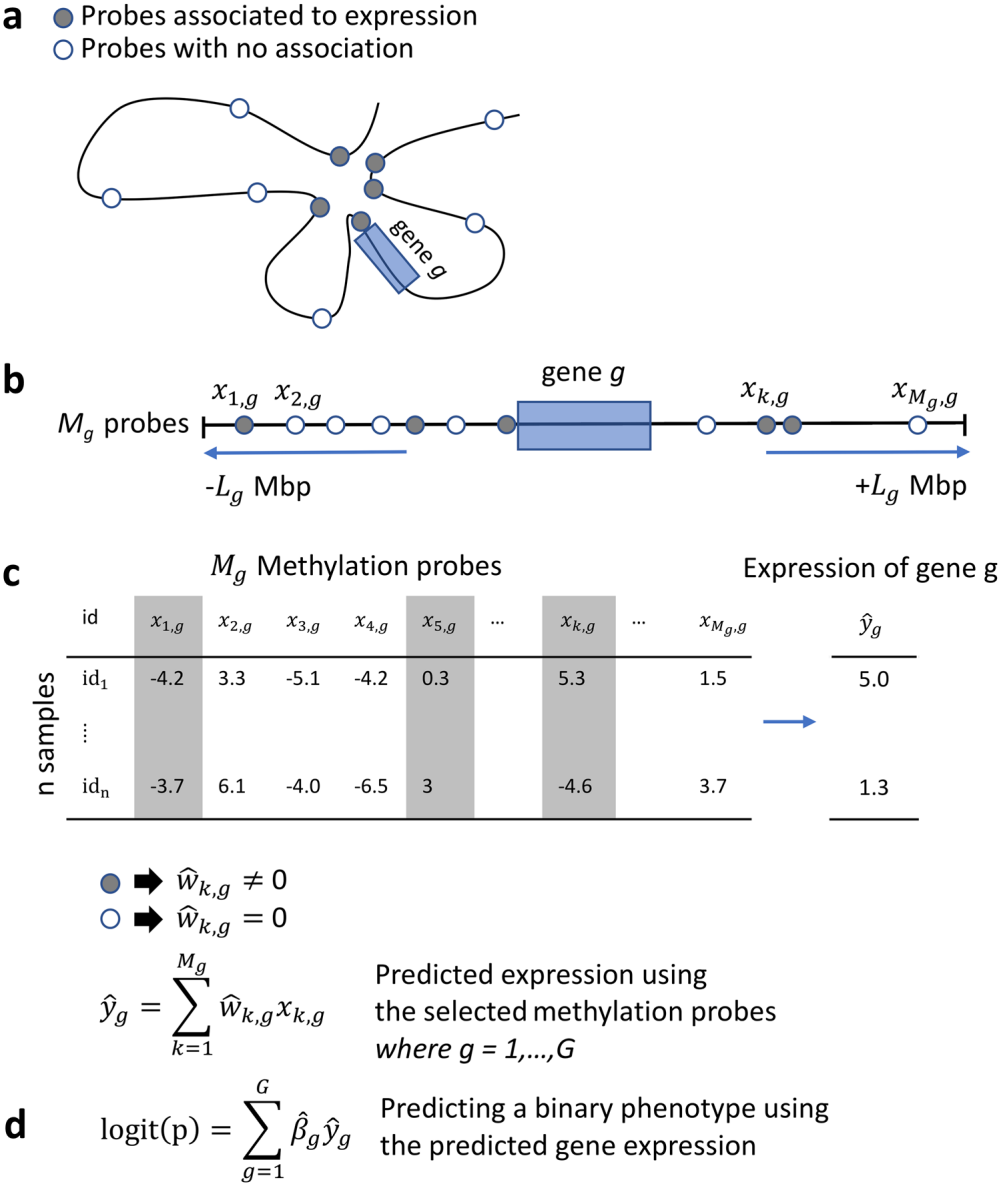
GeneEXPLORE modeling: (**a**) Several methylation probes are associated with gene expression, and they can be located far from the gene due to chromatin looping structure. (**b**) Straightened genome, upstream and downstream ${{L}}_{{g}}$ Mb from the promoter region of the gene g. There are *M*_*g*_ numbers of probes in the range. (**c**) Predicting gene expression from the methylation probes. Methylation data to predict the expression of gene, g consist of n samples and *M*_*g*_ probes. The shaded columns are an example of probes that are associated with gene expression. Our model, geneEXPLORE, identifies the associated probes and estimates the weights of them. Gene expression of g is predicted by summing the weighted methylation values. The procedure is repeated for each gene. (**d**) Application of geneEXPLORE: Predicting phenotypes from the predicted gene expression. After predicting gene expression on the entire genome, we estimated the effects of the predicted gene expression on several binary phenotypes (see Methods).

The training procedure for geneEXPLORE is shown in Fig. 1. First, given a training set of methylation data across samples, we build an elastic-net model^20^, geneEXPLORE, where covariates are long-range methylation probes within a certain distance from the promoter region (*L*_*g*_ in Fig. 1b) and a response is the observed expression level of a gene (Fig. 1c). Elastic-net was chosen because the elastic-net works well in high-dimensional methylation datasets and automatically selects methylation probes that are associated with gene expression.

During the training phase, geneEXPLORE identifies methylation CpG sites that are associated with gene expression and estimate the weights of the identified CpG sites. Second, geneEXPLORE with trained weights is used to predict the gene expression using methylation in the test dataset. Then, we measure the prediction accuracy using R^2^. We repeat the procedure for all genes. Next, using the predicted gene expression by geneEXPLORE as an input, we further build elastic-net logistic regression models to predict binary clinical phenotypes (Fig. 1d). Since we use predicted genes (p = ~14,000) as covariates, instead of methylation probes (p = ~500,000), it is possible to build the prediction model without suffering from high-dimensionality due to the very large number of methylation probes. Through the prediction model, we could estimate the effect of methylation on the phenotypes through gene expression prediction.

### The collective effect of long-range methylation on gene expression is higher than that of promoter and gene region methylation on gene expression

**Figure 2:**
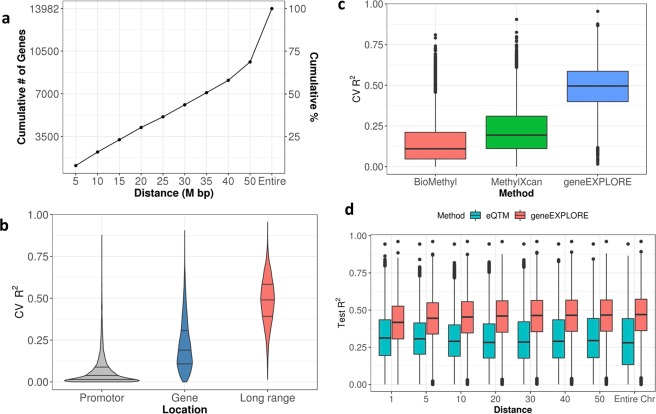
Prediction power (R^2^) comparisons (**a**) Distance (from the promoter region) of probes (L_g_ in *The collective effect of* B) that maximized prediction accuracy (cross-validation (CV) R^2^) for each gene and the cumulative frequency of the genes (y axis on the left) and the percentage (y axis on the right). The distance was selected from ±1 Mb from promoter regions to the entire chromosome on which the gene is located. (**b**) Gene expression prediction power (CV-R^2^) by various regions using TCGA breast cancer data: the predictive models were developed based on methylation probes in (1) the promoter, (2) the gene, and (3) long-range regions. We plotted 13,910 genes for which at least one probe is included in the promoter region of the gene. The three lines in the violin plots indicate 25%, 50%, and 75% percent quantiles, respectively. For long-range regions, the optimal distance was selected for each gene. (**c**) Prediction power (CV R^2^) comparison by methods, MethylXcan, BioMethyl, and geneEXPLORE: Each box plot contains prediction accuracy of 12,114 genes which are predicted by all three methods. (**d**) Prediction power (Test R^2^) comparison: geneEXPLORE vs. multiple regression based on expression quantitative trait methylations (eQTMs) with various distances. Each data point is test R^2^ of each gene. 13,982 genes were predicted.

First, using 13,910 expressed genes in 873 TCGA breast cancer samples, we investigated how the distance of methylation affects gene expression: from ±1 Mb from the promoter region to the entire chromosome on which the gene is located (see Methods). As the associated methylation probes were different for each gene, we selected the distance that maximized prediction accuracy (CV R^2^) (Figs. 2A, S1, S2). For most of the genes, long-range methylation probes were required to predict gene expression accurately: 84% of the genes need methylation probes more than ±10 Mb away to achieve the best prediction accuracy. 49% of the genes required including methylation probes more than ±35 Mb away from the genes to maximize prediction accuracy (Fig. 2A**)**. Also, 31% of the genes required methylation values from the entire chromosome to maximize their gene expression accuracy. This shows that most genes are associated with methylation CpGs that are located more than 10 Mb from the promoters of the genes. A possible reason is that even though most enhancers are within a few Mb from the regulated gene^14^ (also supported by Fig. S1), there can be still several enhancers that are far away (more than 10 Mb).

To understand the methylation effect on various genomic regions, gene expression levels were predicted using methylation probes in 3 different regions: (1) promoter, (2) gene, (3) long-range regions. Gene regions include the promoter region, 5′UTR, first exon, gene body, and 3′UTR as Illumina annotated.

Methylation in long-range predicts gene expression far better (average CV R^2^ = 0.486) than methylation in either promoter (average CV R^2^ = 0.064) or gene regions (average CV R^2^ = 0.218) (Fig. 2b). A possible reason is that the collective effects of *trans*-methylation can exert a stronger effect on gene expression than *cis-*methylation in the promoter or gene region, although individual effects of *trans*-methylation may be weaker than that of *cis*-methylation. These results suggest that distal methylation outside of the promoter and the gene regions can collectively play more important roles in gene expression than methylation on the promoter and the gene regions do.

### geneEXPLORE outperforms state-of-art gene expression prediction methods

To demonstrate the benefit of geneEXPLORE over other comparable modeling tools, we compared geneEXPLORE with other methods. MethylXcan^19^ utilized the lasso method using methylation probes within the gene regions. BioMethyl^18^ used multiple regression method without penalty using pre-selected methylation probes (Pearson’s correlation coefficient >|0.05|) within the gene regions. To compare with BioMethyl, we used R package BioMethyl to get imputed gene expression. We compared CV R^2^ of 12,114 genes, which were predicted by all three methods. geneEXPLORE significantly outperforms the other two methods (Fig. 2c), and BioMethyl performs the worst. (The average CV R^2^ of BioMethyl is 0.148, that of MethylXcan is 0.224, and that of geneEXPLORE is 0.491.)

### Prediction comparison between geneEXPLORE and multiple regression using expression quantitative trait methylations (eQTMs) in TCGA breast cancer

To understand the prediction performance of geneEXPLORE in comparison to a traditional statistical method, geneEXPLORE was compared to a multiple regression model based on eQTMs. We used 75% of the data as training data and 25% of the data as the test data in which prediction accuracy was measured. For eQTMs, methylation probes are selected by univariate tests with Bonferroni correction (p-value <0.05) for each gene, and multiple regression was fitted with the selected probes in training data. We considered methylation probes in various ranges from the promoter region (1, 5, 10, 20,30,40, and 50 Mb, and the entire chromosome on which each gene is located) (see Methods). For all the various distances, geneEXPLORE outperforms the multiple regression model based on eQTMs **(**Fig. 2d). In particular, the difference between the two methods becomes bigger as the distance increases. When methylation probes with the entire chromosome were used, geneEXPLORE predicted 97% of the gene expressions (13,569 out of 13,982) better than eQTMs. A possible reason may be that multiple testing correction methods in eQTMs tend to be too conservative to detect true positives- significant probes that are associated with a gene. Too few true positive probes in the multiple regression models make impossible to predict gene expressions better than geneEXPLORE, which automatically selects probes without statistical tests. This multiple correction issue gets more serious when more probes are tested in broader ranges. This could cause the worse prediction accuracy of eQTMs as the distance increases.

### Testing geneEXPLORE on an independent cohort

**Figure 3:**
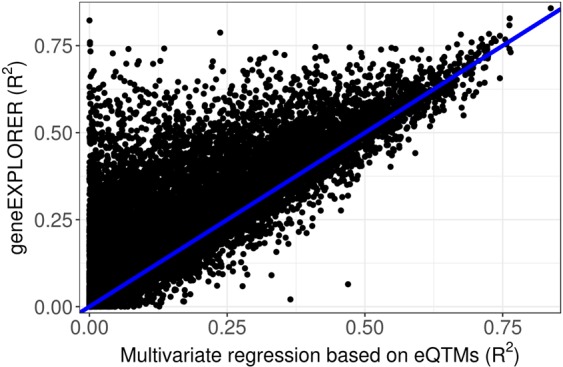
Prediction accuracy on an independent breast cancer cohort (GSE39004). Using the prediction model trained on TCGA breast cancer, prediction accuracy tested on GSE39004 data was compared between geneEXPLORE and a multiple regression based on eQTMs. Methylation probes in ±10 Mb from the promoter regions were used for 13,027 genes.

To show that geneEXPLORE can be used to predict gene expression in an independent cohort, geneEXPLORE trained in the TCGA BRCA was tested on an independent breast cancer cohort. This dataset consists of methylation 450 K array and gene expression microarray datasets of 57 breast tumor samples and 8 adjacent normal samples (GSE39004). The result was compared with that of the multiple regressions based on eQTMs for 13,027 expressed genes. geneEXPLORE significantly outperforms eQTMs in predicting gene expressions of the independent data set (R^2^ = 0.261 for geneEXPLORE and R^2^ = 0.181 for eQTMs on average, t-test p-value = 1.31×10^−312^). We also found that, for a majority of the genes (10,189, 78%), geneEXPLORE predicted gene expression better than the multiple regression based on eQTMs in the independent cohort (Fig. 3), demonstrating its applicability to independent datasets of the same cancer type.

### Applicability of geneEXPLORE to 10 other types of cancer

**Figure 4:**
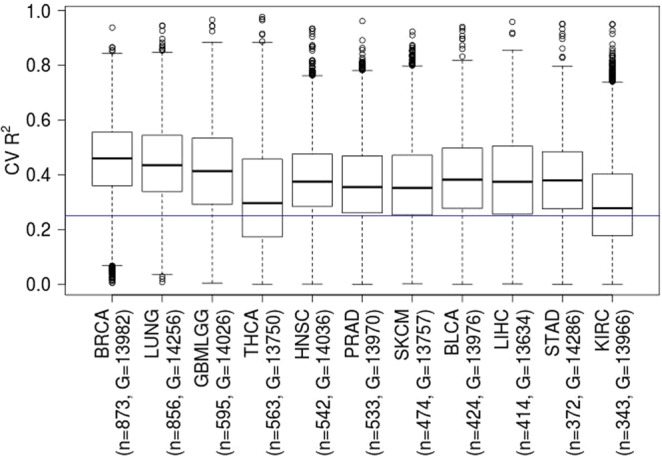
Application of geneEXPLORE to 11 types of cancer data: Breast invasive carcinoma (BRCA), Lung adenocarcinoma and Lung squamous cell carcinoma (LUNG), Glioblastoma multiforme and Glioblastoma multiforme (GBMLGG), Thyroid carcinoma (THCA), Head and Neck squamous cell carcinoma (HNSC), Prostate adenocarcinoma (PRAD), Skin Cutaneous Melanoma (SKCM), Bladder Urothelial Carcinoma (BLCA), Liver hepatocellular carcinoma (LIHC), Stomach adenocarcinoma (STAD), Kidney renal clear cell carcinoma (KIRC). n is the number of samples available in both RNA-seq and 450 K methylation data and G is the number of predicted genes. Each point of a boxplot represents prediction accuracy of each gene measured by cross-validated R^2^. The blue line is at R^2^ = 0.25, which corresponds to the Pearson’s correlation coefficient, r = 0.5.

To demonstrate its applicability to other types of human cancer, in addition to the breast cancer data, geneEXPLORE was applied to 10 other cancer types; Lung adenocarcinoma and Lung squamous cell carcinoma (LUNG), Glioblastoma multiforme and Glioblastoma multiforme (GBMLGG), Thyroid carcinoma (THCA), Head and Neck squamous cell carcinoma (HNSC), Prostate adenocarcinoma (PRAD), Skin Cutaneous Melanoma (SKCM), Bladder Urothelial Carcinoma (BLCA), Liver hepatocellular carcinoma (LIHC), Stomach adenocarcinoma (STAD), Kidney renal clear cell carcinoma (KIRC). We conducted cross-validation to measure prediction accuracy (cv R^2^) for each cancer type. Overall, for the 11 types of cancer, geneEXPLORE shows high prediction accuracy (Median of Pearson’s correlation coefficient, r >0.5, R^2^ >0.25) (Fig. 4). For 9 types of cancers, at least 75% of genes were predicted with high accuracy (r > 0.5). This demonstrated that geneEXPLORE can be applied to other cancer types to predict gene expression in the presence of methylation data.

### geneEXPLORE accurately predicts expression of tumor-associated genes

**Figure 5:**
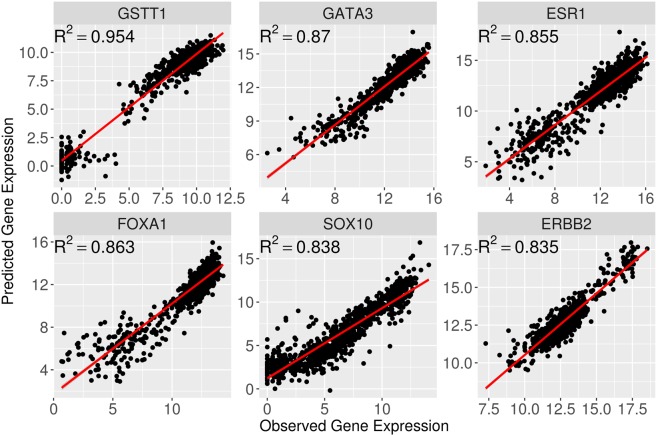
Examples of highly predicted genes that are associated with Breast cancer. The genes were predicted by geneEXPLORE using TCGA breast cancer data. R^2^ is Cross-validation prediction accuracy. The optimal distance was chosen for each gene.

We found that geneEXPLORE accurately predicts the expressions of multiple genes that play important roles in breast cancer. Examples are shown in Fig. 5. Polymorphisms of GSTT1, the highest predicted gene, are established risk factors for breast cancer^21–23^. The mutation of GATA3 is known to lead to luminal tumors^24^. ESR1 is the estrogen-receptor gene, common in primary breast cancers, whose mutation is indicative of resistance to anti-estrogen therapies^25–30^. In addition, breast cancer risk–associated SNPs are enriched in the cistromes of FOXA1 and ESR1^31^. High expression of SOX10 is observed in triple-negative and metaplastic breast carcinomas^32^. ERBB2 is a well-known oncogene of breast cancer^33^.

In addition, we also found that geneEXPLORE predicted many oncogenes and tumor suppressor genes with high prediction accuracy (Table 1). This means that those genes are associated with multiple long-range methylation CpGs. Since many abnormal enhancer activities are found in cancer and enhancer regions are often hypomethylated^13^, the oncogenic mechanism involving the oncogenes and tumor suppressor genes can be associated with abnormal activities in methylation. The roles of these genes in breast cancer have been widely studied at the genetic or transcriptomic level but not as much in epigenetics. Since methylation through long-range interactions predicted a substantial part of gene expression, geneEXPLORE can further help to discover the tumorigenic role of long-range methylation in human cancer.

**Table 1:** Best predicted 20 oncogenes and 20 tumor suppressor genes by geneEXPLORE.

Gene	Full name	Chr.	Distance^b^	CV R^2^
**(a) Oncogene**^**a**^				
*ERBB2*	erb-b2 receptor tyrosine kinase 2	chr17	23	0.835
*VANGL2*	VANGL planar cell polarity protein 2	chr1	entire	0.831
*BCL2*	BCL2, apoptosis regulator	chr18	50	0.792
*CACNA1H*	calcium voltage-gated channel subunit alpha1 H	chr16	10	0.775
*ETV6*	ETS variant 6	chr12	entire	0.755
*CHRD*	chordin	chr3	24	0.743
*NTN4*	netrin 4	chr12	entire	0.737
*EZH2*	enhancer of zeste 2 polycomb repressive complex 2 subunit	chr7	entire	0.736
*STK32B*	serine/threonine kinase 32B	chr4	entire	0.734
*MFGE8*	milk fat globule-EGF factor 8 protein	chr15	40	0.728
*ERBB3*	erb-b2 receptor tyrosine kinase 3	chr12	entire	0.721
*SELP*	selectin P	chr1	entire	0.72
*TCF7*	transcription factor 7 (T-cell specific, HMG-box)	chr5	40	0.715
*BAMBI*	BMP and activin membrane bound inhibitor	chr10	12	0.711
*SLC9A9*	solute carrier family 9 member A9	chr3	entire	0.71
*PLK2*	polo like kinase 2	chr5	17	0.696
*HLA-DRA*	major histocompatibility complex, class II, DR alpha	chr6	33	0.693
*STIL*	SCL/TAL1 interrupting locus	chr1	19	0.693
*VIM*	vimentin	chr10	entire	0.686
*GJB3*	gap junction protein beta 3	chr1	33	0.685
**(b) Tumor suppressor genes**^**a**^				
*GATA3*	GATA binding protein 3	chr10	entire	0.87
*FOXA1*	forkhead box A1	chr14	28	0.863
*TBC1D10C*	TBC1 domain family member 10C	chr11	22	0.813
*BIN2*	bridging integrator 2	chr12	50	0.755
*INTS4*	integrator complex subunit 4	chr11	7	0.754
*EOMES*	eomesodermin	chr3	entire	0.748
*WWP1*	WW domain containing E3 ubiquitin protein ligase 1	chr8	entire	0.745
*TBX3*	T-box 3	chr12	6	0.74
*ADAM33*	ADAM metallopeptidase domain 33	chr20	34	0.733
*DACH1*	dachshund family transcription factor 1	chr13	50	0.727
*ZFP36L2*	ZFP36 ring finger protein like 2	chr2	19	0.726
*TGFBR2*	transforming growth factor beta receptor 2	chr3	36	0.724
*RNF43*	ring finger protein 43	chr17	22	0.723
*B3GNT5*	UDP-GlcNAc:betaGal beta-1,3-N-acetylglucosaminyltransferase 5	chr3	7	0.718
*LIMCH1*	LIM and calponin homology domains 1	chr4	35	0.711
*RAD21*	RAD21 cohesin complex component	chr8	9	0.711
*MXRA8*	matrix remodeling associated 8	chr1	entire	0.706
*TTK*	TTK protein kinase	chr6	50	0.702
*HDAC2*	histone deacetylase 2	chr6	50	0.701
*MARCKSL1*	MARCKS like 1	chr1	entire	0.697

^a^Oncogene and tumor suppressor genes were identified using TUSON algorithm^40^ using the same method as Park *et al*.^41^. ^b^Distance refers to the distance (Mb) from promoter regions to maximize prediction accuracy. Entire refers to the entire chromosome on which the gene is located. CV R^2^ is the squared correlation between the predicted expression and the observed expression using 10-fold cross validation.

### geneEXPLORE accurately predicts clinical features of human cancer based on the predicted gene expressions

Since gene expression profiles often reflect clinical phenotypes^34^, to determine potential clinical applications of geneEXPLORE, we built predictive models using the predicted gene expressions to predict clinical phenotypes of TCGA breast cancer data (see Methods). Based on the predicted expression levels of 13,982 genes, we predicted cancer status (tumor/normal), Estrogen Receptor (ER) status (positive/negative), 5-year survival (yes/no) and PAM50 breast cancer subtypes. Due to the high prediction accuracy of the breast cancer-related genes, high prediction accuracies of these phenotypes were expected.

**Figure 6:**
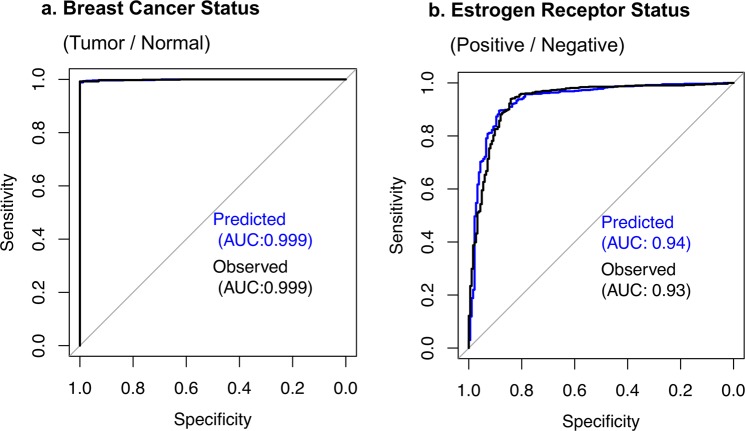
ROC curve for predicting clinical phenotypes using the gene expression predicted by geneEXPLORE (predicted) vs. observed gene expressions (observed*)*: the predicted gene expression predicts the phenotypes as good as the observed gene expressions with perfect prediction accuracy. The gene expression was predicted using methylation probes within 10 Mb from the promoter region of each gene.

Consistent with the expectation, by comparing prediction accuracy between the model using the predicted gene expressions and the model using the observed gene expression, we found that virtually no difference between the predicted gene expressions and the observed gene expressions in predicting the phenotypes (Figs. 6 and Table S1**)**. Notably, gene expression predicted by methylation almost perfectly predicted both cancer status and ER status (AUC = 0.999 and 0.94 respectively) (Fig. 6**)**.

Since the predicted gene expression was the portion of gene expression associated with long-range methylation, the high prediction accuracy of the clinical features implies that many long-range methylation CpGs are highly associated with the phenotypes through gene expressions in breast cancer. This shows that the predicted gene expression can be applied to help diagnose cancer phenotypes or develop personalized treatments as was the approach using observed gene expressions^35^, even when gene expression data are not available.

## Discussion

In this paper, we developed a statistical machine learning model, geneEXPLORE, to quantify methylation effects on gene expression. geneEXPLORE incorporates both *cis-* and *trans-* methylation CpG sites into the statistical model and measures the methylation effect of not only a single CpG site but also the collective effects of long-range CpG sites. Applying geneEXPLORE to the TCGA breast cancer dataset demonstrated that (1) most genes are associated with methylation more than 10 Mb from promoter regions; (2) long-range methylation is highly associated with gene expression, far greater than the effect of methylation in the promoter regions or gene body regions; (3) geneEXPLORE outperforms multiple regression models based on eQTMs for the most highly expressed genes in TCGA breast cancer datasets as well as an independent cohort; (4) many highly predicted genes are related to breast cancer, such as oncogenes and tumor suppressor genes; (5) the predicted gene expression predicted breast cancer status and estrogen receptor status with almost perfect prediction accuracy, where the predicted gene expression and the observed gene expression predicted the phenotypes equally well.

geneEXPLORE was partly motivated by Gamazon *et al*.^36^ who predicted gene expression using SNPs nearby to the genes. However, their models showed a markedly lower prediction accuracy than geneEXPLORE (mean CV R^2^ = 0.137 vs mean CV R^2^ = 0.486). The lower accuracy could be due to smaller effects of SNPs as opposed to larger effects of methylation on gene expression, smaller genomic regions considered (1 Mb from TSS), or different tissue and disease types. Also, Gamazon *et al*. did not directly use the predicted gene expression levels to predict phenotypes. Rather, they developed a method called prediXcan to test the association between the predicted gene expression and several phenotypes. In this study, we used the predicted gene expression to predict clinical phenotypes, showing strong effects of methylation on phenotypes through gene expression.

geneEXPLORE outperforms MethylXcan^19^ and BioMethyl^18^ in terms of gene expression prediction accuracy. The reason can be (1) geneEXPLORE uses the best statistical methods among the three, which is the elastic-net. The elastic-net outperforms the lasso, which MethylXcan uses (Fig. S6). The lasso performs better than the multiple regression, which BioMethyl uses, in predicting gene expression^19^. (2) geneEXPLORE incorporates long-range methylation probes while the other two methods only incorporate methylation probes in gene regions.

geneEXPLORE could not be tested on an independent dataset with the same platform on which it was trained – geneEXPLORE was trained using RNA-seq data but it was tested using gene expression array data (Fig. 3). The reason is publicly available datasets with 450 K methylation array and RNA sequencing in breast cancer were not available with a sufficient sample size. Since we found only a dataset with 450 K methylation array and gene expression array for breast cancer patients (GSE39004), we tested geneEXPLORE on this dataset. This showed worse prediction accuracy than when it was tested within the RNA-seq data (RNA-seq: R^2^ = 0.444 vs microarray: R^2^ = 0.263; Fig. S3), maybe due to the difference between array data and sequencing data, in addition to fitting bias between the training set and the test set.

We showed the applicability of geneEXPLORE in the other 10 cancer types (Fig. 4). Each model was trained and tested in the same cancer type. The prediction accuracy of gene expression was generally high (Pearson’s correlation >0.5) for all the cancer types. This implies that geneEXPLORE method can be applied to other types of cancer. However, one caution is that the model should be trained in a cancer-specific manner as we showed in Fig. S4 since enhancers are cancer-specific^13^. In terms of training sample size required, according to our sub-sampling experiments in TCGA breast cancer data, we found that the model with n = 250 reaches saturation point in terms of prediction accuracy (Fig. S5). We found that among 21 cancer types in TCGA, 16 cancer types have samples around n = 250 or more for both methylation and expression (Table S2). These various types of TCGA cancer data can be served as training data sets. This survey shows that geneEXPLORE is widely applicable to various cancer types.

While we found that selecting optimal distance for each gene within which methylation probes are included for prediction, it takes lots of time to train the model since a user needs to train the model with various distances for entire genes. Rather, we found that predicting gene expression using 10 Mb achieves comparable prediction accuracy as that using optimal distance (Fig. S1). Therefore, we used 10 Mb for various comparisons.

The scope of this study was limited to predicting gene expression and not identifying/discovering regulatory elements such as enhancers. However, since geneEXPLORE selects CpG sites that are associated with gene expressions, the selected CpG sites could be in enhancer or insulator regions. Therefore, geneEXPLORE may be further developed to identify regulatory regions with stability selection approaches^35^.

In conclusion, we developed geneEXPLORE, which predicts gene expression using *cis-* and *trans*-methylation. To the best of our knowledge, geneEXPLORE is one of the first to estimate the collective both *cis-* and *trans*-effects of methylation on gene expression. Using geneEXPLORE, we found that the collective *trans*-effects are greater than the *cis-*effects of methylation. geneEXPLORE predicted about half of gene expression variations on average, which was far more accurate than the estimation using genetic variants from Gamazon *et al*.^36^. In addition, the predicted gene expression successfully predicted cancer phenotypes such as cancer and ER receptor status as accurate as the observed gene expressions. Given these results, future application of geneEXPLORE can be (1) imputation of gene expression for other cancer types or other diseases, (2) discovery of regulatory elements, and (3) prediction of disease/phenotypes.

## Methods

### DNA methylation and RNA sequencing data from TCGA breast cancer

To predict gene expression from methylation data, we analyzed TCGA breast cancer data for 873 samples, whose 450 K methylation array data and Hi-Seq. 2000 gene expression data were available. Among these samples, 788 samples are tumor and 85 samples are normal. The two datasets were downloaded from Xena Public Data Hubs.

### Pre-processing

The values of methylation data in the data hubs are beta values. We transferred beta values to M values because M values are more suitable (closer to normal distributions) for linear regression. The transformation from beta values to the M values to the following: M_i_ = log_2_(beta_i_/(1 - beta_i_)).

Among 485,577 probes, we removed 90,007 methylation probes whose values were missing in more than 20% of the samples. Then, we imputed 31,700 methylation probes whose missing rates were less than 20% using K-means clustering (R package REMP).

For gene expression data, among 20,530 genes, we excluded 3,417 genes whose average expression levels are less than 1 (RPKM) from the prediction. Among the 17,113 genes, TSS sites are available for 16,681 genes from UCSC genome browser. Among these, there was at least one probe in promoter regions for 13,982 genes. We included these genes in our final analysis.

geneEXPLORE (gene expression prediction by long-range epigenetics)

In detail, for each gene, we built a linear regression model to predict gene expression using long-range methylation probes.

1 $${\hat{y}}_{g}=\mathop{\sum }\limits_{k=1}^{{M}_{g}}{\hat{w}}_{k,g}{x}_{k,g}$$

where ${\hat{y}}_{g}$ is the predicted expression of gene *g*, ${x}_{k,g}$ is k-th methylation probe for gene *g*, ${\hat{w}}_{k,g}$ is the regression coefficient of the methylation probe, ${M}_{g}$ is the number of methylation probes within a defined region (e.g. 10 Mb or the entire chromosome). To estimate the weight ${\hat{w}}_{k,g}$, we used the elastic-net penalty^18^ with α = 0.5 (the combination of half Lasso and half ridge penalty) and the penalty was selected through cross-validation using the R package glmnet. The implementation of geneEXPLORE is done through R, and available in https://github.com/SoyeonKimStat/geneEXPLORER.

Elastic-net was chosen to predict gene expression using long-range methylations for the following reasons. First, the elastic-net works well with a high-dimensional methylation dataset. Up to ~38,000 probes were included in the model while the number of samples was only 873. It is impossible to accurately predict gene expression using such high dimensional data using a model based on regular linear regression models without penalization. Second, the elastic-net automatically selects important variables that are associated with a response. By utilizing the elastic-net, geneEXPLORE automatically selects methylation probes that are associated with gene expression from tens of thousands of methylation probes and builds gene expression prediction models based on the probes. Third, the elastic-net works well in highly correlated datasets^18,35,36^. Since some of the methylation values are highly correlated due to biological interactions, in our analysis, the elastic-net works better than Lasso for 86% of the genes (Fig. S6).

### Measuring prediction accuracy

To measure prediction accuracy, 10-fold cross-validation (CV) was used. 9 folds of data were used to build a model. The model used methylation values in the remaining fold to predict gene expression. We repeated the procedure 10 times until all gene expressions were predicted. For 81 patients, more than 2 samples existed for the same patient in the dataset (79 patients – 2 samples, 2 patients – 3 samples). We assigned the samples for the same patients to the same fold to avoid bias. Prediction accuracy (R^2^) was measured as the squared Pearson’s correlation coefficient between predicted gene expression and true gene expression.

### Comparing the prediction accuracy of different regions in a gene

We defined a promoter region from 2000 bp upstream and 0bp downstream of the transcription start site of a gene^37^. Gene regions were obtained using R packages IlluminaHumanMethylation450kanno.ilmn12.hg19, which is annotated by Illumina. The gene regions include promoter region, 5′UTR, first exon, gene body, and 3′UTR.

The long-range regions refer to the regions that maximize prediction accuracy using geneEXPLORE. The range is from ±1 Mb from the promoter region to the entire chromosome on which the gene is located. We fitted the elastic-net model (Eq. 1) for each region and each gene. We showed prediction accuracy of 13,910 overlapping genes (among 13,982 genes) for which all the following conditions were satisfied; (a) Gene region was available in the R package (b) there was at least one probe in the promoter region.

### Investigation of various distances from promoter regions of genes

For each gene, we built elastic-net models using methylation CpG sites for various distances (1, 2, …, 10, 20, 30, 40, 50 Mb) from the promoter region of the gene. The elastic-net model was also built using all CpG sites on the same chromosome where the gene is located. Prediction accuracy was evaluated using 10-fold CV R^2^. Then, distances were selected that maximized the prediction accuracy for each gene.

### Evaluating prediction accuracy using multiple regression based on eQTMs

Since traditional multiple regression cannot handle high dimensional data (the number of samples <the number of probes), methylation probes were pre-screened before fitting multiple regression models. For each gene, an association between a gene and each methylation probe was tested using single linear regression models, where the covariate is a probe and the response is a gene (expression). To fast computing, we adapted matrixeQTL package^38^. A multiple-testing adjustment was performed for each gene using Bonferroni correction at significance level 0.05. Using the significantly associated probes, we built a multiple linear regression model for each gene. If the significantly associated genes were still more than the number of samples in a training set, a ridge regression model^39^ was fitted. We used 75% of the data for training and 25% of the data for the testing data set.

### Testing on an independent cohort

geneEXPLORE was trained using TCGA breast cancer data and tested on GSE39004 data.

geneEXPLORE models were built using TCGA data for each gene, and models were selected that minimized CV error using 10-fold CV. Using the methylation probes from the test dataset as inputs of the models, gene expression was predicted for 13,027 genes. Test R^2^, which is squared Pearson’s correlation coefficient between the predicted gene expression and the observed gene expression of the test dataset, was calculated.

For comparison, multiple regression models based on eQTMs were used, as in the previous section. We used the training data to select significant probes, using univariate tests with Bonferroni correction ($\alpha =0.05)$ and to fit multiple regression models. Using the methylation array data in the test data as input to the models, gene expression was predicted using the multiple regression model for each gene, and test prediction accuracy was calculated. We limited long-range distance to 10 Mb from promoter regions to save computational time.

### Predicting clinical phenotypes

Breast cancer status and estrogen receptor (ER) status were predicted using the predicted gene expression. For cancer status, 788 samples were tumor and 85 samples were normal, among 873 samples from the TCGA breast cancer data. For ER status, 632 samples had ER-positive status,183 samples had ER-negative status, while 58 samples had missing ER status.

To predict the clinical phenotypes, 13,982 gene expressions were first predicted in test datasets in the same cohort. The data was divided into a training set (4/5 of the samples) and a test set (1/5 of the samples). Using the training dataset, 10 folds cross-validation (4/50 of samples are in each fold) was used to select a model that maximized prediction accuracy using probes within ±10 Mb from the promoter regions. By inputting methylation in the test dataset into the selected model, gene expression in the test dataset was predicted. The procedure was repeated five times until all gene expression data was predicted.

Next, a penalized logistic regression model (elastic-net) was fitted using the 13,982 gene expressions as covariates, and a phenotype as a binary response, as described in the following equation:

2 $$logit(p)=\mathop{\sum }\limits_{g=1}^{G}{\hat{\beta }}_{g}{\hat{y}}_{g}$$

where *p* is the probability of a phenotype to be “Yes” (e.g. tumor/ER-positive), ${\hat{y}}_{g}$ is the predicted expression of gene *g*, ${\hat{\beta }}_{g}$ is the regression coefficient of gene *g*, and *G* is the number of predicted genes (13,982).

Note that the elastic-net model automatically selects gene expression that is associated with the phenotype. Prediction accuracy was evaluated by area under the ROC curve (AUC) using 10-folds CV.

## Electronic supplementary material

Supplementary information.

## Data Availability

USCS genome browser https://genome.ucsc.edu/. TCGA breast cancer data from UCSC XENA https://xenabrowser.net/datapages/?cohort=TCGA%20Breast%20Cancer%20(BRCA)&removeHub=https%3A%2F%2Fxena.treehouse.gi.ucsc.edu%3A443. TCGA lung cancer data from UCSC XENA. https://xenabrowser.net/datapages/?cohort=TCGA%20Lung%20Cancer%20(LUNG)&removeHub=https%3A%2F%2Fxena.treehouse.gi.ucsc.edu%3A443. Gene expression omnibus GSE39004 dataset. https://www.ncbi.nlm.nih.gov/geo/query/acc.cgi?acc=GSE39004.

## Code availability

**geneEXPLORE is freely available in the GitHub repository**.

https://github.com/SoyeonKimStat/geneEXPLORER.

**R packages**.

**BioMethyl** (ver 1.1) https://github.com/yuewangpanda/BioMethyl.

**REMP** (ver 1.2.5) https://bioconductor.org/packages/release/bioc/html/REMP.html.

**glmnet** (ver 2.0.18) https://cran.r-project.org/web/packages/glmnet/index.html.

**IlluminaHumanMethylation450kanno.ilmn12.hg19** (ver 0.6.0) http://bioconductor.org/packages/release/data/annotation/html/IlluminaHumanMethylation450kanno.ilmn12.hg19.html.

## References

[1] Jones PA (2012). Functions of DNA methylation: islands, start sites, gene bodies and beyond. Nat Rev Genet.

[2] Zemach A, McDaniel IE, Silva P, Zilberman D (2010). Genome-wide evolutionary analysis of eukaryotic DNA methylation. Science.

[3] Razin A, Cedar H (1991). DNA methylation and gene expression. Microbiol Rev.

[4] Shen H, Laird PW (2013). Interplay between the cancer genome and epigenome. Cell.

[5] Stadler MB (2011). DNA-binding factors shape the mouse methylome at distal regulatory regions. Nature.

[6] Gutierrez-Arcelus M (2013). Passive and active DNA methylation and the interplay with genetic variation in gene regulation. Elife.

[7] Gutierrez-Arcelus M (2015). Tissue-specific effects of genetic and epigenetic variation on gene regulation and splicing. PLoS Genet.

[8] Irizarry Rafael A, Ladd-Acosta Christine, Wen Bo, Wu Zhijin, Montano Carolina, Onyango Patrick, Cui Hengmi, Gabo Kevin, Rongione Michael, Webster Maree, Ji Hong, Potash James B, Sabunciyan Sarven, Feinberg Andrew P (2009). The human colon cancer methylome shows similar hypo- and hypermethylation at conserved tissue-specific CpG island shores. Nature Genetics.

[9] Kulis, M. & Esteller, M. In *Advances in Genetics* Vol. 70 (eds Zdenko Herceg & Toshikazu Ushijima) 27–56 (Academic Press, 2010).

[10] Ehrlich M (2009). DNA hypomethylation in cancer cells. Epigenomics.

[11] Aran D, Sabato S, Hellman A (2013). DNA methylation of distal regulatory sites characterizes dysregulation of cancer genes. Genome Biol.

[12] Yao L, Shen H, Laird PW, Farnham PJ, Berman BP (2015). Inferring regulatory element landscapes and transcription factor networks from cancer methylomes. Genome Biol.

[13] Sur I, Taipale J (2016). The role of enhancers in cancer. Nat Rev Cancer.

[14] Mora A, Sandve GK, Gabrielsen OS, Eskeland R (2016). In the loop: promoter-enhancer interactions and bioinformatics. Brief Bioinform.

[15] Herranz Daniel, Ambesi-Impiombato Alberto, Palomero Teresa, Schnell Stephanie A, Belver Laura, Wendorff Agnieszka A, Xu Luyao, Castillo-Martin Mireia, Llobet-Navás David, Cordon-Cardo Carlos, Clappier Emmanuelle, Soulier Jean, Ferrando Adolfo A (2014). A NOTCH1-driven MYC enhancer promotes T cell development, transformation and acute lymphoblastic leukemia. Nature Medicine.

[16] Beagrie Robert A., Scialdone Antonio, Schueler Markus, Kraemer Dorothee C. A., Chotalia Mita, Xie Sheila Q., Barbieri Mariano, de Santiago Inês, Lavitas Liron-Mark, Branco Miguel R., Fraser James, Dostie Josée, Game Laurence, Dillon Niall, Edwards Paul A. W., Nicodemi Mario, Pombo Ana (2017). Complex multi-enhancer contacts captured by genome architecture mapping. Nature.

[17] Ron G, Globerson Y, Moran D, Kaplan T (2017). Promoter-enhancer interactions identified from Hi-C data using probabilistic models and hierarchical topological domains. Nature Communications.

[18] Wang Y, Franks JM, Whitfield ML, Cheng C (2019). BioMethyl: an R package for biological interpretation of DNA methylation data. Bioinformatics.

[19] Zhong H, Kim S, Zhi D, Cui X (2019). Predicting gene expression using DNA methylation in three human populations. PeerJ.

[20] Zou H, Hastie T (2005). Regularization and Variable Selection via the Elastic Net. Journal of the Royal Statistical Society. Series B (Statistical Methodology).

[21] Gudmundsdottir K, Tryggvadottir L, Eyfjord JE (2001). GSTM1, GSTT1, and GSTP1 genotypes in relation to breast cancer risk and frequency of mutations in the p53 gene. Cancer Epidemiol Biomarkers Prev.

[22] de Aguiar ES (2012). GSTM1, GSTT1, and GSTP1 polymorphisms, breast cancer risk factors and mammographic density in women submitted to breast cancer screening. Rev Bras Epidemiol.

[23] Xiao ZS, Li Y, Guan YL, Li JG (2015). GSTT1 polymorphism and breast cancer risk in the Chinese population: an updated meta-analysis and review. Int J Clin Exp Med.

[24] Takaku M, Grimm SA, Wade PA (2015). GATA3 in Breast Cancer: Tumor Suppressor or Oncogene?. Gene Expr.

[25] Jeselsohn R (2014). Emergence of constitutively active estrogen receptor-alpha mutations in pretreated advanced estrogen receptor-positive breast cancer. Clin Cancer Res.

[26] Merenbakh-Lamin K (2013). D538G mutation in estrogen receptor-alpha: A novel mechanism for acquired endocrine resistance in breast cancer. Cancer Res.

[27] Nadji M, Gomez-Fernandez C, Ganjei-Azar P, Morales AR (2005). Immunohistochemistry of estrogen and progesterone receptors reconsidered: experience with 5,993 breast cancers. Am J Clin Pathol.

[28] Rhodes A, Jasani B, Balaton AJ, Barnes DM, Miller KD (2000). Frequency of oestrogen and progesterone receptor positivity by immunohistochemical analysis in 7016 breast carcinomas: correlation with patient age, assay sensitivity, threshold value, and mammographic screening. J Clin Pathol.

[29] Robinson DR (2013). Activating ESR1 mutations in hormone-resistant metastatic breast cancer. Nat Genet.

[30] Toy W (2013). ESR1 ligand-binding domain mutations in hormone-resistant breast cancer. Nat Genet.

[31] Cowper-Sal lari R (2012). Breast cancer risk-associated SNPs modulate the affinity of chromatin for FOXA1 and alter gene expression. Nat Genet.

[32] Cimino-Mathews A (2013). Neural crest transcription factor Sox10 is preferentially expressed in triple-negative and metaplastic breast carcinomas. Hum Pathol.

[33] Revillion F, Bonneterre J, Peyrat JP (1998). ERBB2 oncogene in human breast cancer and its clinical significance. Eur J Cancer.

[34] Bao T, Davidson NE (2008). Gene expression profiling of breast cancer. Adv Surg.

[35] Kim S, Baladandayuthapani V, Lee JJ (2017). Prediction-Oriented Marker Selection (PROMISE): With Application to High-Dimensional Regression. Stat Biosci.

[36] Gamazon ER (2015). A gene-based association method for mapping traits using reference transcriptome data. Nat Genet.

[37] Dozmorov MG, Cara LR, Giles CB, Wren JD (2012). GenomeRunner: automating genome exploration. Bioinformatics.

[38] Shabalin AA (2012). Matrix eQTL: ultra fast eQTL analysis via large matrix operations. Bioinformatics (Oxford, England).

[39] Friedman J, Hastie T, Tibshirani R (2010). Regularization Paths for Generalized Linear Models via Coordinate Descent. J Stat Softw.

[40] Davoli T (2013). Cumulative Haploinsufficiency and Triplosensitivity Drive Aneuploidy Patterns and Shape the Cancer Genome. Cell.

[41] Park Hyun Jung, Ji Ping, Kim Soyeon, Xia Zheng, Rodriguez Benjamin, Li Lei, Su Jianzhong, Chen Kaifu, Masamha Chioniso P., Baillat David, Fontes-Garfias Camila R., Shyu Ann-Bin, Neilson Joel R., Wagner Eric J., Li Wei (2018). 3′ UTR shortening represses tumor-suppressor genes in trans by disrupting ceRNA crosstalk. Nature Genetics.

