# Supplementary material for: Collective effects of long-range DNA methylations predict gene expressions and estimate phenotypes in cancer
Source: Sci Rep. 2020 Mar 3;10:3920. doi: 10.1038/s41598-020-60845-2 (PMC7054398; doi:10.1038/s41598-020-60845-2)
Supplement: Supplementary file 1 — Supplementary information. [file 41598_2020_60845_MOESM1_ESM.pdf]

SUPPLEMENTARY DATA

Collective effects of long-range DNA methylations predict gene expressions and estimate phenotypes in cancer

Soyeon Kim[1,2], Hyun Jung Park[3], Xiangqin Cui[4], Degui Zhi[5,*]
[1]Department of Pediatrics, School of Medicine, University of Pittsburgh, Pittsburgh, Pennsylvania, United States
[2]Division of Pediatric Pulmonary Medicine, UPMC Children's hospital of Pittsburgh, Pittsburgh, Pennsylvania, United States
[3] Department of Human Genetics, Graduate School of Public Health, University of Pittsburgh, Pennsylvania, United States
[4] Department of Biostatistics and Bioinformatics, Emory University, Atlanta, Georgia, United States
[5] School of Biomedical Informatics, University of Texas Health Center at Houston, Houston, Texas, United States

*To whom correspondence should be addressed. Email: Degui.Zhi@uth.tmc.edu

Comparison of prediction accuracy using geneEXPLORER between NGS and microarray platforms

We compared the performance of gene expression prediction between next generation sequencing (NGS) and microarray, even though geneEXPLORER was trained using gene expression using NGS (**Figure S3**). For NGS prediction, since a breast cancer dataset for which both NGS and 450K methylation array was not available, TCGA breast cancer data was divided into training (4/5 of the samples) and test (1/5 of the samples) datasets. A model was trained and selected using CV (using methylation probes within 10Mb from promoter regions) within the training set and prediction accuracy of test dataset was measured. The procedure was repeated 5 times until all samples were imputed. For microarray prediction, geneEXPLORER was trained using the TCGA breast cancer dataset and tested on another breast cancer dataset (GSE39004), which had both 450K methylation array and microarray gene expression.

As expected, geneEXPLORER predicted NGS gene expression much better than microarray gene expression**:** on average, test R_2 of NGS was 0.444 while test R_2 of microarray is 0.263. However, it was also able to predict gene expression with moderate prediction accuracy (the average correlation coefficient was 0.514).

Cancer specificity of geneEXPLORER

Since enhancers are cancer specific, we expected that geneEXPLORER models are cancer specific as well. To demonstrate this, we applied geneEXPLORER trained in TCGA breast cancer data to predict 13,823 genes of TCGA lung cancer data. The results demonstrated that the model did not work in lung cancer (mean R_2=0.02), confirming our hypothesis (**Figure S4**).

Predicting additional phenotypes using predicted gene expression

In addition to cancer status and ER status, 5 years survival and breast cancer sub-type were also predicted. For survival data, since 732 samples (83.8%) were censored among 873 patients, there were only 298 samples whose 5 year-survival data are available. 207 patients died before 5 years and 91 patients lived more than 5 years. If censoring occurred before 5 year of follow-up, 5-year survival is indicated as NA. If censoring occurred after 5 year of follow-up, 5-year survival is indicated as Yes. The model predicted 5 years survival with lower accuracy (AUC=0.71) than cancer status or ER status, possibly due to the high portion of censored data (**Figure S5**).

The breast cancer sub-type status was available for 620 samples. The subtypes are Luminal A (LumA), Luminal B (LumB), Triple-negative/basal-like (Basal), HER2-enriched (Her2), and Normal-like (Normal). Using the predicted gene expression, breast cancer subtypes were accurately predicted with 0.174 mis-classification error. Using observed (true) gene expression, breast cancer subtype was predicted with similar prediction accuracy with 0.134 mis-classification error (**Table S1**).

Training sample size calculation

We investigate gene expression prediction accuracies using geneEXPLORE with various training sample sizes (50, 100, 150, .., 650) and tested on 221 samples from TCGA breast cancer data. As we tested 3 genes, we found that the model with n=250 reaches saturation point in terms of prediction accuracy as shown in Figure S6. We also conducted a survey that shows many samples are available in TCGA data in various cancer types for methylation (array) and Expression (sequencing) (**Table S2**). We found that among 21 cancer types, 16 cancer types have samples around n=250 or more for both methylation and expression. These various types of TCGA cancer data can be served as

training data sets. This survey shows that geneEXPLORE is widely applicable for various cancer types

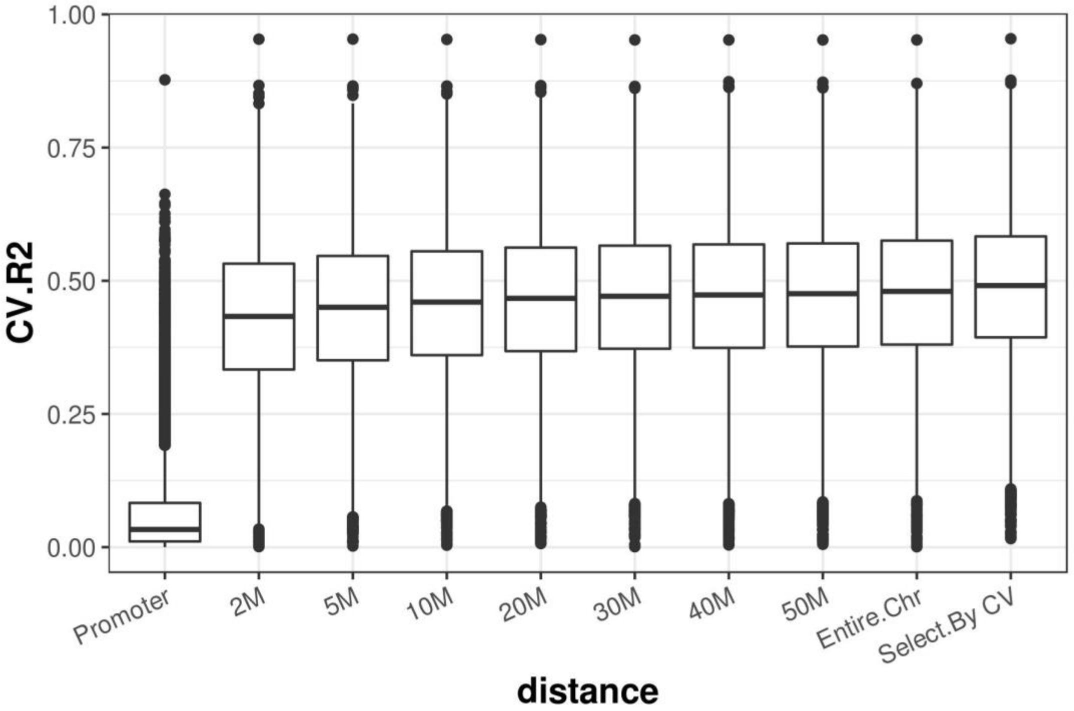

Figure S1. Prediction accuracy of gene expression (Cross-validated R_2) with various **dist ance from promoter regions**: *We included* probes within *promoter regions,* 2Mb, 5Mb,10Mb, 20Mb,30Mb,40Mb, and 50Mb from promoter regions as inputs for the model. Finally, *we inclu ded* all probes in the entire chromosome on which the gene located *(referred as Entire.Chr)*. F urther, we selected the distance which maximized prediction accuracy by CV for each gene a nd included all probes within the distance as inputs for the model (referred as Selected.By CV). The number of genes included in the boxplot is 13,910.

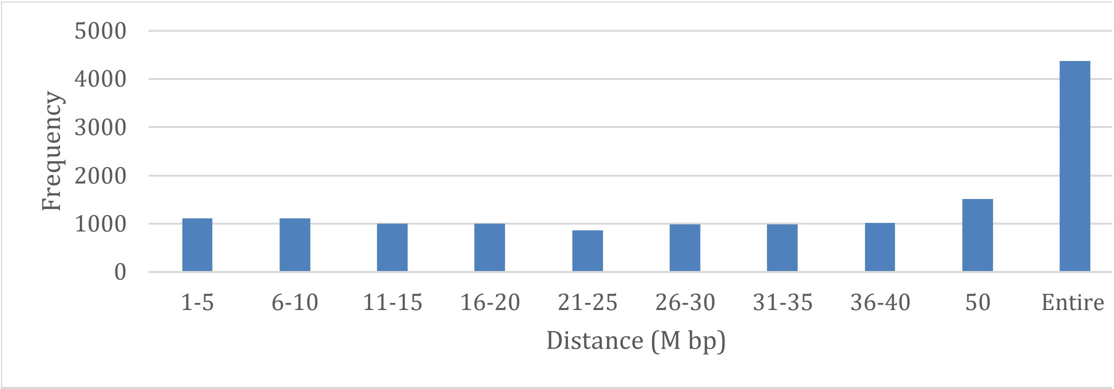

Figure S2. The distances from promoters which predict gene expression the best: frequency is the number of genes. To maximize prediction accuracy, most genes require inclusion of methylation very distant to the genes.

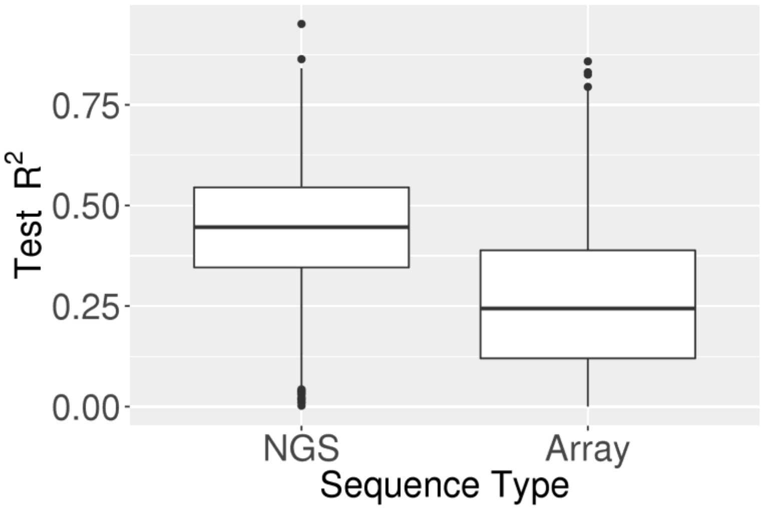

Figure S3. **Comparison of prediction accuracy using geneEXPLORER between NGS and microarray platforms**: geneEXPLORER trained on TCGA breast cancer and tested prediction accuracy of gene expression on gene expression data using NGS methods (Left) and using microarray method (Right) The results demonstrate prediction accuracy of 10,972 overlapping genes in both datasets.

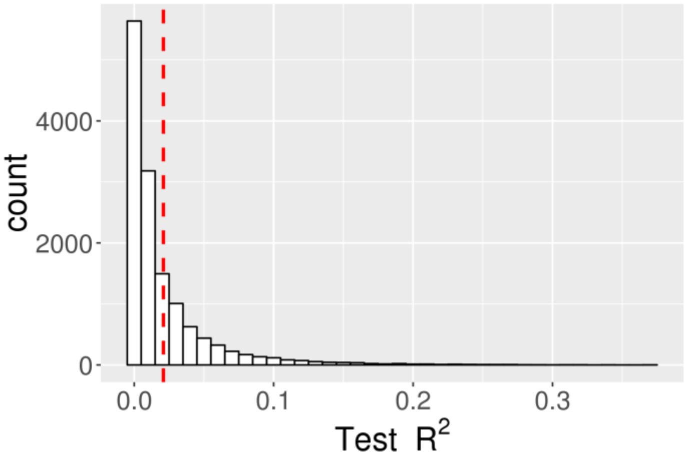

Figure S4. Cancer specificity of geneEXPLORER. When gene EXPLORER trained on breast cancer was tested in lung cancer, it showed low prediction accuracy.

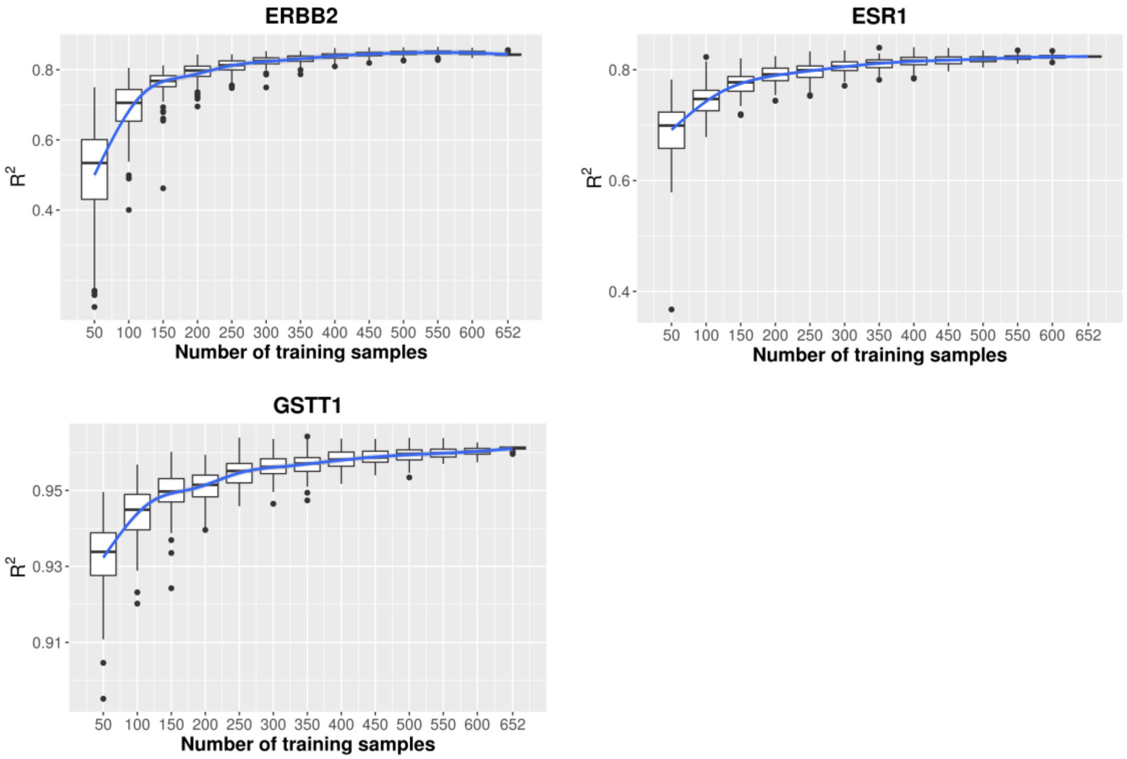

Figure S5. Prediction power (R_2) of gene expression using geneEXPLORE developed from training data with various sample sizes (n=50, 100, 150, …,600, and 652) and testing data with ¼ of the entire data (n=221) (TCGA breast cancer) for (A) ERBB2 (B) ESR1 (C) GSTT1. 100 random samples were conducted for each training sample size. R_2 is saturated around n=250. Probes within 10Mb from promoter regions were used to build the models.

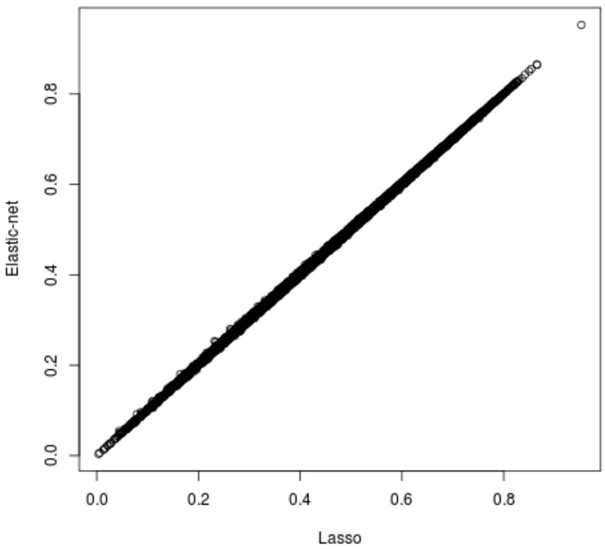

Figure S 6. Comparison of cross-validated R2 between Lasso vs Elastic Net to predict gene expression using methylation probes within 10Mb from promoter regions for 13982 genes from TCGA breast cancer data. Pearson's correlation between two is 0.99991. The prediction accuracy using the elastic net is better than the lasso for 86.18% of the genes.

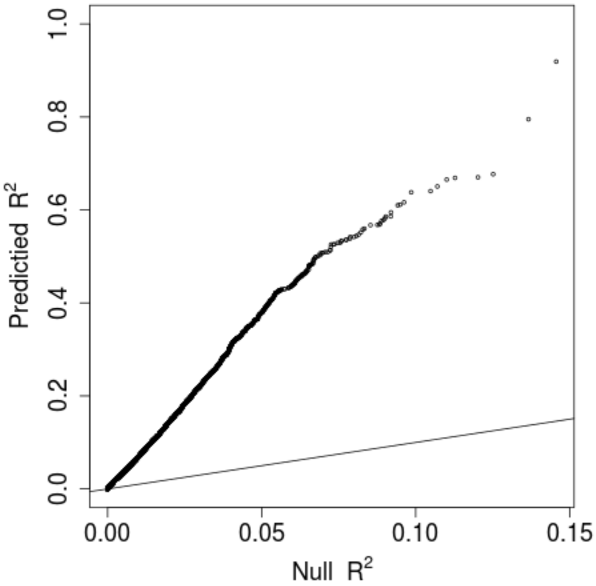

Figure S 7. Prediction performance of geneEXPLORE tested on a separate cohort. Using geneEXPLORE trained in TCGA Lung cancer data set using methylation array and RNA seq, we tested the model in another lung cancer data (GSE60645). The data consists of 117 samples for which both methylation array data and gene expression array data available. Methylation probes within 10Mb from the promoter region of each gene were used to predict gene expression. Gene expression data were only used to measure prediction accuracy. The data are publicly available in gene expression omnibus. In the qqplot, R_2, between predicted and observed expression levels plotted against the null distribution of R_2.

Table S1. Confusion Matrix: Predicting PAM50 breast cancer sub-type using (a) gene

		Predicted						Predicted				
		Basal	Her2	Lum A	Lum B	Normal		Basal	Her2	Lum A	Lum B	Normal
True	Basal	84	1	1	0	1	Basal	85	1	0	1	0
	Her2	3	22	0	6	0	Her2	0	22	1	8	0
	Lum A	0	0	252	27	9	Lum A	0	1	260	17	10
	Lum B	0	0	49	78	0	Lum B	0	1	33	93	0
	Normal	2	1	8	0	76	Normal	2	1	7	0	77

A. Predicted gene expression
Misclassification error: 0.174

B. Observed gene expression
Mis-classification error: 0.134

expression predicted by geneEXPLORER (b) Observed gene expression

Table S 2. The number of samples available in TCGA data in various cancer types for methylation (array) and Expression (sequencing).

Project Name	Primary Site	Methylation (Array)	Expression (Sequencing)
Breast Cancer	Breast	1013	1041
Brain Glioblastoma Multiforme	Brain	404	159
Ovarian Serous Cystadenocarcinoma	Ovary	581	262
Uterine Corpus Endometrial Carcinoma	Uterus	513	508
Kidney Renal Clear Cell Carcinoma	Kidney	513	518
Head and Neck Squamous Cell Carcinoma	Head and neck	494	481
Lung Adenocarcinoma	Lung	481	478
Brain Lower Grade Glioma	Brain	438	439
Head and Neck Thyroid Carcinoma	Head and neck	502	500
Lung Squamous Cell Carcinoma	Lung	427	428
Prostate Adenocarcinoma	Prostate	358	375
Skin Cutaneous melanoma	Skin	430	430
Colon Adenocarcinoma	Colorectal	424	428
Gastric Adenocarcinoma	Stomach	443	418
Bladder Urothelial Cancer	Bladder	273	295
Liver Hepatocellular carcinoma	Liver	243	294
Cervical Squamous Cell Carcinoma	Cervix	243	259
Kidney Renal Papillary Cell Carcinoma	Kidney	216	222
Acute Myeloid Leukemia	Blood	194	173
Pancreatic Cancer	Pancreas	124	142
Rectum Adenocarcinoma	Colorectal	153	154

The information is available at ICGC data portal https://dcc.icgc.org/
